# Supplementary material for: Active Pharmacovigilance Study: A Follow-Up Model of Oral Anti-Cancer Drugs under Additional Monitoring
Source: Curr Oncol. 2023 Apr 11;30(4):4139–52. doi: 10.3390/curroncol30040315 (PMC10137106; doi:10.3390/curroncol30040315)
Supplement: Supplementary file 1 [file curroncol-30-00315-s001.zip › curroncol-2267176-supplementary.pdf]

Table S1. Chemical structures and suspected toxicities of the studied oral antineoplastics.

Medicines under additional monitoring	Chemical Structure[1]	Suspected toxicity
Alectinib	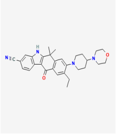	• Gastrointestinal disorders<br>• Liver disorders<br>• Lung disorders<br>• Kidney disorders
Cabozantinib	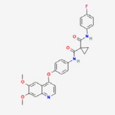	• Mucosites<br>• Hypertension
Entrectinib	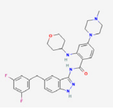	• Hypotension<br>• Vomiting
Ixazomib	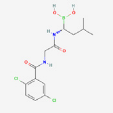	• Haematological disorders
Lenalidomide	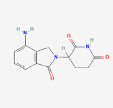	• Anorexia<br>• Haematological disorders<br>• Neurological disorders
Lorlatinib	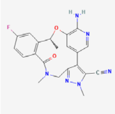	• Dyslipidemia
Niraparib	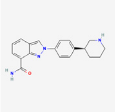	-
Osimertinib	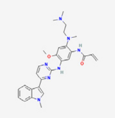	• Paronychia
Palbociclib	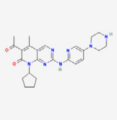	• Haematological disorders
Ribociclib	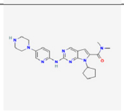	• Haematological disorders
Trametinib + Dabrafenib	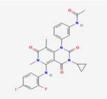 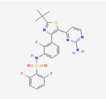	• Fever
Vandetanib	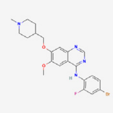	• QT prolongation<br>• Skin disorders
Venetoclax	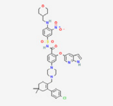	• Haematological disorders
Trifluridine + tipiracil	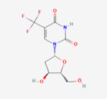 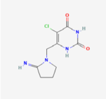	• Gastrointestinal disorders<br>• Haematological disorders<br>• Proteinuria

[1]All the chemical structures images were retrieved from the website https://pubchem.ncbi.nlm.nih.gov/. [Accessed 25 Mar 2023].
